# On the Analysis of Sweat, the Acid Contained in It, and the Acids of Urine and Milk

**Published:** 1808-02

**Authors:** 

**Affiliations:** Member of the National Institute, &c.


### *On the Analysis of Sweat, the Acid contained in it, and the Acids of Urine and Milk.* By Mr. THENARD, Member of the National Institute, &c.

ON examining the principal fluids of the animal body, we discover that some are alkaline, while others are acid. Under the first class, are comprehended the blood and bile; and, under the second, the urine, milk, and sweat. Hence two questions naturally arise; 1. What are the alkalies? and, 2. What are the acids peculiar to these different fluids?

The researches of Cadet and Deyeux, have already solved the first, by demonstrating that soda is the only alkali present in animal substances. Little progress has, however, been made towards a solution of the second; even the data that might lead to this desirable object are, for the most part, inaccurate; and many of the results relating to some of them, are too deficient in proof, to be admitted as demonstrated truths. It is this second question, therefore, which we propose to consider in the present Paper; but, in order to treat it in a proper manner, it will be first necessary to exhibit as full and complete an analysis of the sweat, as has been given of the urine and milk.

Sweat is a fluid, separated from the blood in the skin by exhalant vessels, with which its texture is furnished. It is, more or less, abundant in different individuals; and its quantity is in the inverse ratio of that of the urine. Other circumstances being equal, much more is produced during digestion, than during a state of absolute rest. The maximum of its production appears to be twenty-six grains and two-thirds in a minute; the minimum nine grains troy. It is, however, much inferior to the pulmonary transpiration; and there is also a considerable difference between their nature, and the manner of their formation. The one is the product of a particular secretion, in a certain degree similar to that of the urine; the other, which is composed of a great portion of water and carbonic acid, is the product of a combustion, slowly effected by atmospheric air.

The sweat, in a healthy state, very sensibly reddens litmus paper or infusion. In certain diseases, and especially in putrid fevers, it is alkaline; yet its taste is always rather saline, and similar to that of salt than acid. Though colourless, it stains linen. It has a peculiar smell, which almost becomes insupportable, when it is concentrated; which is particularly the case during distillation. But before mentioning the trials to which I subjected it, and for which I required a considerable quantity, I ought first to mention the method I took to procure it.

I applied to individuals, who are in the habit of wearing flannel-waistcoats next the skin. To obviate every source of fallacy before being put on, they were well washed with soap; then rinsed in running water, and afterwards several times in diluted muriatic acid; and, lastly, they were immersed, and wrung out of a large tub of water. The persons who obligingly submitted to this experiment, previously went into a bath, and had every part of their body well rubbed. The sweat which collected in the flannel, during the course of ten days, I separated by means of hot distilled water; and this I boiled down to the consistence of a syrup in a retort, to the neck of which a receiver was fitted. The product of this distillation exhaled a nauseous smell; which, however, diminished as the liquor cooled. It produced no alteration in syrup of violets; but reddened, in a very sensible degree, the infusion of litmus. After being some time exposed to the action of the air, it still retained its original transparency, and suffered no remarkable change, except in regard to the smell, which wholely disappeared. In a close vessel, it probably would have putrified; like the products of the distillation of other animal fluids.

The residuum was wholely free from smell, and not very abundant; though strongly acid, the agreeable taste of sea salt predominated; it nevertheless, however, left an acrid and pungent sensation in the mouth; it was only slightly deliquescent, and required the lapse of a few days, before it passed into a liquid state. It was completely soluble in water.

Lime, barytes, ammonia, the acidulous oxalate of potash, the carbonates of pot-ash and soda, most acids, and the acetate of lead, when added to this solution, produced no precipitate with it, nor disengaged any thing from it. Nut-galls occasioned a slight precipitate in it, and the nitrate of silver rendered it extremely turbid.

It was decomposed on being calcined, by itself emitting vapours, which partook not, however, of the fetid odour of animal matter, and became converted into a black substance; which I found to be composed of a great quantity of common salt, charcoal, and a very minute portion of lime, and of oxide of iron.

On

On being subjected to calcination, after the acid was saturated with pot-ash, this base was obtained in the state of a carbonate; beside the preceding matters contained in the remaining black substance.

From these trials, I was convinced that sweat contains muriate of soda, some traces of phosphate of lime, and oxide of iron; a small portion of animal matter, no sulphate, no soluble phosphate, but an acid; the nature of which I began to conjecture.

In fact, this acid, combined with a base, producing a carbonate by calcination, must evidently belong to the vegetable or animal kingdom; and as, moreover, it was volatile, and formed soluble salts, with the different salifiable bases, it became extremely probable that it was the acetous acid.

Induced by this reasoning, to conjecture that the acetous acid was present in sweat, I deemed it necessary to enter on some direct experiments, with the view of ascertaining the truth of this conjecture; for though the properties above-mentioned belong only to the acetous of all the known acids, yet they might equally appertain to an unknown acid. Thus azote is far from being sufficiently characterised by the properties with which we usually rest satisfied, as denoting its presence, viz. its being wholely destitute of smell and colour, as well as from its exerting no sensible action on blue colours, or solution of lime; since all these properties are negative, and consequently less discriminative than those, which being founded on combinations, may be termed positive. In order to afford complete certainty, it is farther necessary that there be a combination of these positive properties; unless some one of them, as is the case in certain instances, be so decisive, as to afford a satisfactory test of its presence.

Thus, though a concurrence of circumstances tended to evince that it was the acetous acid which was present in sweat, it was, nevertheless, necessary to obtain it separately, and combine it with different substances, before venturing to pronounce certainly with respect to its nature. This I readily effected, by distilling, with another acid, the residuum, which a certain quantity of sweat, collected in a flannel waistcoat, slightly alkaline, afforded by evaporation. In this process I preferred the phosphoric acid, not only from its being fixed, but because being extremely difficult to decompose, it exerts less action on organic matters than most others. I, besides, took the farther precaution of condensing, as much as possible, the product duct of distillation in the receiver. This product, which had the taste of a weak acid, imparted a deep red to the infusion of litmus; it exhaled an odour like that of vinegar; combined with pot-ash, it formed a salt, which, by evaporation, was reduced to shining scales, resembling mica, acrid, and very deliquescent. On the addition of sulphuric or phosphoric acid to this salt, a strong smell of acetic acid was evolved; and when poured into a solution of nitrate of mercury, it precipitated crystalline scales, similar to acetite of mercury.

This then was the acetous acid; from which it results, that human sweat is composed of a great deal of water, free acetous acid, muriate of soda, a very small portion of phosphate of lime and oxide of iron, and an extremely minute quantity of animal matter, which more nearly resembles gelatine than any other substance.

Urine contains, first, the uric acid, which frequently gives rise to the stone in the bladder; secondly, the benzoic acid, which exists very rarely in that of adults, or old persons, but is very frequent in that of infants; thirdly, since urine always sensibly reddens tincture of litmus, we are compelled to admit the presence of another acid; as this action cannot be ascribed either to the uric acid, which produces no change in the colour of that tincture, or to the benzoic acid, which is only found in the urine under certain circumstances, that have not hitherto been sufficiently ascertained.

What this new acid may be, is the second question, which I shall attempt to examine. At present, it is generally supposed to be the phosphoric. This opinion is founded on the presence of a considerable quantity of the phosphate of lime in urine, which, being itself insoluble when neutral, becomes very soluble, and even deliquescent, when it is combined with an excess of acid. This supposition is farther strengthened by the consideration, that besides the phosphates of lime, soda, ammonia, and magnesia, we find nothing in urine but the sulphates of pot-ash and soda, and the muriates of soda and ammonia, neither of which salts is decomposed by the acidulous phosphate of lime; the sulphuric and muriatic acids cannot, therefore, exist in the urine, since, as it is well known, they would convert the phosphate of lime into acidulous phosphate of lime. If, then, the phosphoric acid be not the solvent of the phosphate of lime in urine, it must unquestionably be some other weak acid; and, most probably, probably, one partaking of the nature of the vegetable and animal acids.

Nothing, in fact, proves this supposition to be erroneous. I will even venture to add, that this hypothesis appears to me more admissible than the former; for, to admit the acidulous phosphate of lime in urine, it is necessary to suppose, that a portion of one of the phosphates of the blood is decomposed in the kidneys, as soon as it reaches them; that the phosphoric acid is free, or, at least, constitutes an acidulous phosphate, with the phosphate of lime, though present with the soda of the blood, and with the base of the decomposed phosphate; both of which appear not to enter into any new combination at the time, and which are taken up with the residuum of the secretion, by the venous system, to be returned into the circulation.

It may be observed with truth, that living bodies act in a different manner from what they do when deprived of vitality; and that, consequently, decompositions may take place in the animal economy, contrary to our experience. But besides that this answer, though accurate, proves little in favour of the case in question, it may be employed, in a certain degree, to impugn the above argument. We have no positive instance of salts being decomposed in the animal economy, so that their acid and alkali remain present together, without entering into a state of combination; while, on the other hand, it is demonstrated, that animal substances, and more especially those which exist in the blood, as, for example, the fibrine and albumen are transformed into some other, in passing thro' different organs; thus, in the mammary glands, they are converted into sugar of milk, and the caseous, butyraceous and extractive matters; and in the kidneys they form urea, uric acid, and sometimes benzoic acid. Now if they uniformly form one of these acids, and sometimes the other also, it is possible they may form a third, which, combining with the phosphate of lime, holds it in solution. Such were the considerations, which induced me to examine the acid of urine; and I shall now proceed to relate the experiments which I made, with the view of discovering its true nature.

After having employed several means, which proved unsuccessful, at least directly, I evaporated nearly to dryness, in a water bath, that I might not decompose the urea, about twenty quarts of recent urine. The residuum imparted imparted a deep red colour to the infusion of litmus; and I several times treated it cold, with a great portion of alcohol, at 36° of strength.

In this way I succeeded in dissolving the greatest part of the acid; but I could not effect its complete solution, whatever quantity of alcohol I employed, even when assisted by a small degree of heat. Having mixed all the liquors, I concentrated them by evaporation, at a low temperature. I then examined the matter, which I had thus reduced to the consistence of syrup. I first diluted a portion of it with water, after which I added to it lime-water and ammonia. No precipitate took place; or at least, it was so slight, as not to be perceptible, till a considerable time after the mixture was made. Another portion I subjected to the process of calcination. The residuum was not acid; and even when treated with water, the calcareous salts and lime-water, added to the solution, gave no indication of the presence of the smallest portion of phosphate. That which was not dissolved, and which contained a great proportion of coal on being completely incinerated, merely left a few traces of phosphate of lime.

From these experiments it should seem, that besides the uric acid, urine contains an acid with, at least, a binary radical. I was much inclined to suspect that it was the acetous, as I had already found this acid present in other animal fluids; it exists in most vegetables, and is formed in almost the whole of the decompositions, which organised bodies undergo. Hence I was induced to add barytes water to the portion I had left, containing the acid. Having then evaporated the mixture to dryness, by means of a gentle heat, I again treated it with alcohol, which dissolved the whole, except a yellowish powder, which proved to be true acetate of barytes. From this experiment we may therefore infer, that urine contains acetous acid; though it does not prove the absence of phosphoric acid, since urine, evaporated by a water-bath, and treated with a large quantity of alcohol, uniformly leaves a slightly acid residuum; and this acid, it may be said, is the phosphoric.

I could not avail myself of calcination, to prove that this acid is not the phosphoric; since the residuum, containing phosphate of ammonia, must have yielded phosphoric acid: I was, therefore, necessarily constrained to adopt the synthetical method. With this view, after having saturated with pot-ash the extract of some urine, which I had evaporated to dryness, with the precautions

 aboveabove-mentioned, I poured in a little vinegar, treated it with alcohol, and obtained the same results as I have already noticed; or in other words, the portion that was not dissolved after repeated effusions of alcohol was acid. I am fully aware that this proof may still be called in question, since, if the phosphoric acid was present in the urine, it would be partly retained by the salts existing therein, in the same way as the acetous, and would become insoluble in alcohol. If, however, it be considered that the presence of the acetous acid in urine seems certain*; that nothing proves the existence of the phosphoric; that the greater part of the free acid of the urine evaporated to the consistence of a syrup is soluble in alcohol; and that all this acid, thus dissolved, is the acetous. Lastly, If we call to mind, that the residuum is slightly acid, and that when saturated by means of pot-ash, afterwards acidulated with vinegar, and treated again with alcohol, it continues equally acid. All these circumstances, when compared together, warrant us to conclude that it is the acetous acid alone in urine which dissolves the calcareous phosphate, and which alone most frequently imparts to it the property of reddening the infusion of litmus.

In order to render this last conclusion more evident, it is necessary to demonstrate still more directly than has hitherto been done, that the benzoic acid is not, in fact, a constant principle of urine. With this view, instead of employing sublimation with or without an excess of another acid, when the urine is reduced to the consistence of syrup, which is always an inaccurate method, since the benzoic acid combined with ammonia is carried off in a greater or less quantity with the water that ascends in the form of vapour: before commencing the evaporation, I added a certain portion of lime, and treated the extract with alcohol. It must be acknowledged, that besides the benzoate of lime, there are dissolved by this method some urea, muriate of ammonia, soda, and acetous acid; but if the alcoholic solution be converted into a concentrated aqueous solution, the acids afterwards added will soon render

* It appears to me, that, in the evaporation of the urine in a water bath, a little urea is decomposed, and that ammonia, and perhaps a small portion of acetous acid is formed. But admitting this to be the case, it is still extremely probable, that the acid of urine is the acetous acid, and not any other; for in support of this opinion, I might not only adduce the reasons that have already been, or will be given, but even the tendency which in this case the urea would have to be changed into acetous acid.

der manifest the smallest portion of benzoic acid which may be present in the solution.

Thus in order accurately to analyse urine, the first object ought to be to discover the presence of benzoic acid, either by this or a similar process. If by such means we are unable to discover the slightest trace of it in the liquid, which is usually the case, we may safely conclude that it does not contain it in any sensible quantity; then having evaporated another portion of the urine, in a water-bath, and by this means ascertained the quantity of water which enters into its composition, the residuum ought to be treated repeatedly with alcohol at 36°; in this way the urea is dissolved as well as the muriate of ammonia, some muriate of soda, and the greater part of the acetous acid. The mixture of these different substances ought to be divided into three separate portions. From the first the acetous acid must be separated by the means already pointed out. From the second the urea must be extracted by concentrated nitric acid, from which it must be afterwards separated by means of the carbonate of pot-ash and alcohol*. And lastly, the quantity of sal ammoniac and muriate of soda contained in the third portion is to be ascertained by sublimation. During this process the urea is destroyed, the acetous acid volatilized, while the muriate of soda remains behind, the weight of which should be ascertained. The sal ammoniac sublimes, and ought to be collected; but as it is uniformly mixed with black matters, and may also contain a little carbonate of ammonia, it must be purified by dissolving it in water, and evaporating the solution.

The different matters contained in urine, which are soluble in alcohol, are five in number; viz. acetous acid, benzoic acid, muriate of ammonia, a portion of the muriate of soda, and urea. Those which are insoluble in it are more numerous, being eight in number; viz. four phosphates, two sulphates, muriate of soda, and uric acid. These eight substances, though insoluble in alcohol, may be partly dissolved by treating them with water. In this number may be included the phosphates of soda and ammonia, a very little phosphate of magnesia, the muriate of

 soda,

* Pure urea is not crystallizable, it is only when in a state of combination with certain salts, which frequently ocuurs, that it shoots into crystals. It appears to me, that it renders several salts soluble in alcohol, which by themselves resist its action. This might satisfactorily be ascertained with muriate of barytes.

soda, the sulphates of pot-ash and soda, which are evident by their crystallization, and which may be separated from each other in some measure by solutions of platina. We may ascertain that phosphate of magnesia is present, by the employment of pot-ash, which will precipitate a small quantity of this earth.

The substances insoluble in water are, therefore, the phosphate of lime, some phosphate of magnesia, combined with phosphate of ammonia and uric acid, which may be separated in the usual manner. This method differs however very little from that given by other chemists; and I shall describe it here in as concise a manner as possible, because it is intimately connected with my subject.

As milk immediately on being drawn from the mammary glands, reddens litmus paper, it is hence evident that it contains a free acid. On discovering this fact, about eighteen months ago, I vainly endeavoured to find it pure, with the view of examining its properties; and my most strenuous endeavours since that period, to attain the same object, have proved equally abortive.

While every thing tends to confirm the belief that this is the acetous acid, we yet remain in the same situation in regard to it, as to the acids of sweat and urine. To form a decisive judgment respecting its true nature, we must first succeed in obtaining it separate, and afterwards combine it with salifiable bases. This I at length effected by pursuing a method similar to that by which I succeeded in obtaining the acid of urine.

1st. I evaporated the milk to dryness.

2dly, I treated the residuum with barytes water, in order to saturate the acid.

3dly, I again evaporated the solution to dryness.

4thly, I treated the evaporated mass with alcohol, to partly dissolve the extractive matter, and particularly to collect the caseous substance, so that none of it might remain suspended in the water.

5thly, I macerated in water the portion which remained undissolved by the alcohol, after which I filtered the liquor, then concentrated it by evaporation, and, lastly, distilled it with phosphoric acid. By these means I collected in the receiver, a fluid possessing all the properties of acetous acid.

From the various experiments I have described, it follows: 1st, That urine probably does not contain any free phosphoric acid; but that there is present in it, as well as in milk and sweat, acetous acid. 2dly, That, besides this this acid, the sweat contains a great quantity of water, some muriate of soda, a small portion of animal matter, and some traces of oxide of iron and phosphate of lime.

It is highly probable that the acetous acid, likewise, exists in several other substances. Various observations lead me to believe, that it will be found in cantharides; the analogy of the bombic and formic acids with vinegar, has already been suspected; and to me it seems highly probable, that it exists in most animals, as in the sap of most vegetables; at least we may venture to affirm, that of all the acids, its formation is the most common in nature. Its principles have such a tendency to enter into union with each other, that it is almost impossible to disturb the equilibrium of the molecules of organised substances, without producing it in a greater or less quantity. Whether the decomposition be rapid or slow, still acetous acid is formed; as is shewn by the distillation of vegetable and animal substances, their treatment by nitric and by oxygenised muriatic acid, their spontaneous decomposition, and their conversion into vegetable mould, or adipocere.

It is well known, in cases of indigestion, that the food becomes acid by the generation of acetous acid. Under several circumstances, however, its formation has not been sufficiently ascertained; it still remains to be proved, whether it exists in the milk of every species of animals; whether it be present in the sweat of all, and whether the sweat of different animals be identical; and, lastly, whether it is present in the state of acetate in alkaline urine.

